# jChIP: a graphical environment for exploratory ChIP-Seq data analysis

**DOI:** 10.1186/1756-0500-7-676

**Published:** 2014-09-26

**Authors:** Krzysztof Chojnowski, Krzysztof Goryca, Tymon Rubel, Michal Mikula

**Affiliations:** Department of Genetics, Maria Sklodowska-Curie Memorial Cancer Center and Institute of Oncology, Warsaw, 02-781 Poland; Institute of Radioelectronics, Warsaw University of Technology, Warsaw, 00-665 Poland

**Keywords:** Next-generation sequencing, Computational genomics, Data analysis, ChIP-Seq

## Abstract

**Background:**

Chromatin immunoprecipitation coupled with next-generation sequencing (ChIP-Seq) provides a powerful tool for discovering protein-DNA interactions. Still, the computational analysis of the great amount of ChIP-Seq data generated, involving mapping of raw data to reference genome, has been a bottle neck for most of researchers in the transcriptional and epigenetic fields. Thus, user-friendly ChIP-Seq processing method sare much needed to enable greater community of computational and bench biologists to exploit the power of ChIP-Seq technology .

**Findings:**

jChIP is a graphical tool that was developed to analyze and display ChIP-Seq data. It matches reads to the corresponding loci downloaded from Ensembl Genes or Ensembl Regulation databases. jChIP provides a friendly interface for exploratory analysis of mapped reads as well as peak calling data. The built-in functions for graphical display of reads distribution allows to evaluate the quality and meaning of ChIP-Seq data.

**Conclusion:**

jChIP is a user-friendly GUI-based software for the analysis of ChIP-Seq data within context of known genomic features. Further, jChIP provides tools for discovering new and refining known genome-wide protein binding patterns.

## Findings

### Background

Chromatin immunoprecipitation followed by next generation sequencing (ChIP-Seq) is a technique for the examination of interactions between proteins and DNA on a genome-wide scale [1, 2]. It enables identification of genome regions occupied by chromatin associated proteins and defining functionally important regulatory elements. The output of ChIP-Seq experiments consists of short nucleotide sequences corresponding to the possible binding sites of a given protein. These sequences, called reads, are then mapped to a reference genome, providing a list of genomic interaction sites. The positions data set may then be searched for reads distribution around specific locations or at cis-regulatory elements.

Numerous software tools for the ChIP-Seq data processing have been developed [3–9]. Selected features of these applications are listed in Table 1. For example the UCSC genome browser web-based application can be used to display genomic positions of interest, browse whole genome and compare displayed datasets [7]. However it does not allow to fast batch process of a large number of data files due to transfer constrains. The other types of applications like BEDTools [6], HOMER [3], ChipSeeker [5] or CisGenome [4] can be run locally. Some of these, however, involve a complex installation process or need large local files with indexed databases. As a result they require big disk space, the running process is complicated and time consuming. Some of these applications support only command line interface, so there is no easy way to perform fast and easy analysis for users who are not familiar with these types of operations. Therefore, there is a pressing need for a consistent GUI-driven environment enabling the user to browse, examine and compare results from separate experiments.

**Table 1:** **Comparison of selected software tools for ChIP-Seq data analysis**

Application	Interface	Installation	Platform	Functionality	Pros	Cons
UCSC genome browser [7]	web-based	not needed	platform independent	genome browsing, tracks displaying, tracks comparing, referring to known genomic features	no installation, graphical interface, rich annotation database	batch process of a large number of data files not possible
BEDTools [6]	command-line	compilation from source through package managers	UNIX LINUX MacOS	interrogation and manipulation of genomic features, comparisons of discontinuous features	fast, divided into several applications	no graphical interface,
HOMER [3]	command-line	Perl installation scripts	UNIX LINUX MacOS Cygwin	data visualisation, peak and enriched motif finding, assembling data across multiple experiments, annotating peaks, basic quality control (sequence bias, fragment length estimation), creating histograms, and heatmaps, re-centering peaks on motifs	fast, divided into several applications, multiple additional scripts helpful by analysis	no graphical interface
ChipSeeker [5]	R package	through R package manager	platform independent (R package needed)	data visualisation, peak detection, pathways enrichment analysis, retrieving the nearest genes around the peak, genomic region annotation, peak significance estimation, conservation analysis, clustering analysis, data comparison with GEO database	interaction with other R packages,	R environment required, programming skill needed
CisGenome [4]	GUI (MS Windows only) command line	compilation from source installer (for MS Windows)	packages for all platforms (GUI only for MS Windows)	peak detection, gene annotation, motif analysis, motif mapping, novel motif discovery, data visualisation	GUI (MS Windows only), divided into several applications	no graphical interface (Linux, UNIX, MacOS)
jChIP	GUI	not required	platform independent (Java runtime environment required)	data visualisation, matching reads to genomic locations, datasets comparision, creating reads count histograms, basic quality control	no installation, graphical interface	only exploratory analysis available

Here we present jChIP, a Java-based application that allows for ChIP-Seq data analysis in a convenient graphical form, making it faster and user-friendly. Given that jChIP can run on any workstation it facilitates collaboration between researches. Sharing project files including analysis results and protocols enables further data processing by other team members. This flexibility provides opportunities for closer interactions between computational and bench biologists.

### Implementation

jChIP is fully implemented using version 7 of Java Runtime Environment (JRE). jChIP loads mapped reads from chosen files. Genes and regulatory elements locations are downloaded from Ensembl database using BioMart API [10].

### jChIP description

#### Data import

jChIP is able to handle reads mapped to genome stored in various file formats (default Bowtie output format, SAM, BAM, BED), allowing to import raw data obtained directly from mapping tools, as well as data preprocessed by peak calling algorithm (WIG). Loci, representing coordinates of functional genome regions or other regions of interest, can be downloaded from the Ensembl data-base (Ensembl Genes or Ensembl Regulation) or loaded from a local file in a couple of available formats (BED, GFF, MACS). Features retrieved from the data-base are user defined. These may include features connected with genes: transcription or gene start/end sites, gene lengths, names, and Gene Ontology annotations or features describing regulatory elements: start/end coordinate, length or description. Loci can be further filtered according to gene or feature type, length, Gene Ontology annotation or minimum allowed distance between loci.

### Single experiment analysis

Once imported, reads are automatically assigned to the corresponding loci, taking into account a user-specified tolerance window and each locus coordinates. Two types of filtration are available for assigned data: the first one takes into account the number of reads in a given window around the loci, while the second type is applied to reads count in single-nucleotide genome positions.

Detailed information about all the loci with at least one read assigned can be displayed as table, with columns representing chromosome, locus position, gene ID in which a given locus lies, number of reads, description of gene function and gene length. The tables can be sorted by any of these parameters and exported as tab-delimited text files. A summary report containing information about the number of loaded and filtered loci/reads and data processing parameters can also be generated and saved in HTML format. Furthermore, several ChIP-Seq quality metrics recommended by the ENCODE consortium [11] including the number of unique reads, non-redundant fraction and PCR Bottleneck Coefficient are computed.

jChIP implements several methods of ChIP-Seq data visualization. The first one is the binding profile – the summary distribution of reads over loci. It shows reads count as function of the distance from loci coordinates separately for positive and negative strand. Another type of plot, especially helpful in establishing appropriate filtering parameters for the given experiment, is the histogram of the number of reads assigned to the tolerance windows around loci or to distinct single-nucleotide positions. Finally, an interactive graphical representation of the reads distribution across whole chromosomes can be plotted as a heatmap which represents reads density for individual loci that can be chosen from the list. All the plots generated by jChIP can be exported as PNG images or stored in vector graphics files (SVG, EPS and PDF).

### Multiple experiments analysis

jChIP offers the possibility to compare multiple data sets through simultaneous visualization of binding profiles and histogram plots originating from separate sequencing runs. Furthermore, a scatter plot of the number of reads assigned to the same loci set in two experiments can be plotted. Additional comparative analysis options include: the χ2 test for assessing the statistical significance of the fit between the observed binding profiles and the calculation of the correlation coefficient (Pearson and Spearman) between the number of reads assigned to the common loci set in two experiments. Multi-experimental tables can also be generated, in which case two methods are available: tables may include the sum of all the positions present in the selected experiments, where empty values are filled with zeros, or the intersection of position sets.

### User interface

**Figure 1:**
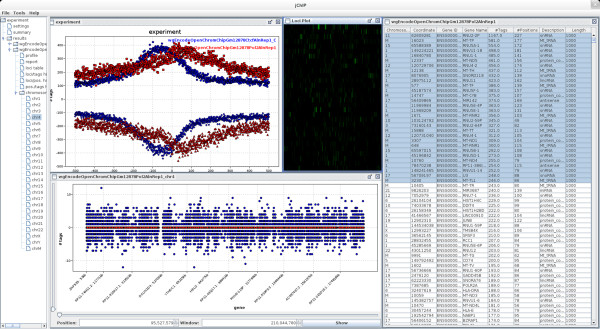
**The main window of jChIP with example analysis results: comparison of two summary binding profiles (top left), table with number of reads assigned to genes (right), visualization of reads distribution around selected genes using heatmap (top middle) and number of reads assigned to each gene (bottom).** Browsable tree containing all currently opened experiments is localized on the left side of the screen.

jChIP is a standalone multi-platform program developed in Java language. It features multi-window graphical environment in which multiple ChIP-Seq experiments can be accessed simultaneously and compared (Figure 1). Furthermore, it provides a batch analysis mode, therefore numerous data sets can be processed at once. The results are stored as text files in a selected experiment directory and are readily available for the user every time jChIP is started. The configuration is saved at application shutdown and automatically restored, so the assays can be continued after jChIP is restarted. All processing parameters can be exported (and loaded) to a single XML file, enabling protocol dissemination.

### Use of jChIP

**Figure 2:**
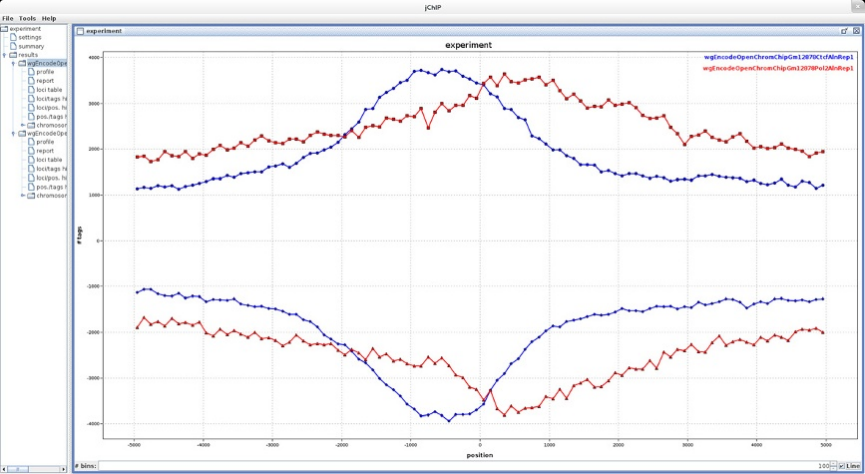
**Common loci profile showing cumulative binding profiles of CTCF and Pol2 relatively to TSS.** Datasets were taken from GEO database (GSM822312 and GSM822270).

To illustrate the jChIP usage we employed two Chip-Seq data sets produced within the frames of ENCODE project [12] for CTCF and Polymerase II RNA (Pol2) proteins and available in the GEO database under GEO-ID GSM822312 and GSM822270, respectively. An example of binding profiles for both factors relatively to the genes transcription start sites (TSSs) coordinates is depicted on Figure 2. The profiles indicate that for Pol2 a cumulative peak of binding is moved into the body of the gene, just below TSS. On the other hand, a peak of CTCF binding is positioned about 500 base pairs upstream of TSS. The list of loci occupied by either CTCF or Pol2 was further inspected in jChIP generated table. Sorting according to the number of aligned reads pointed RNU-2P and IGHIII-38-1 loci as the most abundantly bound by for Pol2 and CTCF, respectively.

For the CTCF datafile (7029958 reads) jChIP needed 2GB of RAM and 18 MB for data storage. The analysis took 40 seconds. Processing of the Pol2 data (16316956 reads) needed 2.5GB of RAM and 20 MB on the hard disk. The processing time was 60 seconds. The analysis was run on AMD Phenom II X6 1090 T 3200 MHz processor with 8GB of RAM.

### Discussion

jChIP provides user-friendly tool for analysis of the data generated by ChIP-Seq. The key features are: 1) Faster mapping of reads to corresponding DNA loci and generating protein-DNA interaction profiles, 2) Information about DNA elements may be downloaded from databases or read from local file using different formats, 3) Partial results of the whole analysis process can be saved to a file allowing application of different computing parameters without repeating all steps multiple times, 4) Results are stored in a local file system so the user has access to them every time jChIP is started, 5) Analysis configuration is stored in a file eliminating the need to have a new set up for different experiments.

### Conclusions

jChIP provides an easy to use graphical environment for fast and user-friendly interrogation of ChIP-Seq data. It facilitates quality assessment generating tables with data statistics and their display. jChIP is written in Java thus it is platform-independent and able to run on desktop computers. It also does not require to store any index files for experiment data processing.

## Availability and requirements

**Project name:** jChIP

**Project home page:**http://sourceforge.net/projects/jchip

**Operating systems:** Platform independent

**Programming language:** Java

**Other requirements:** Java 1.7 or higher

**License:** GNU GPL

**Any restrictions to use by non-academics:** No restrictions

Availability of supporting data

The data sets supporting the results of this article are available in the GEO repository: GSM822312, GSM822270.

## Abbreviations

Chip-Seq: Chromatin immunoprecipitation followed by next generation sequencing
TSS: Transcription start site
Pol2: Polymerase II RNA.
